# Septoplasty versus non-surgical management for nasal obstruction in adults with a deviated septum: economic evaluation alongside a randomized controlled trial

**DOI:** 10.1186/s12916-020-01562-5

**Published:** 2020-05-01

**Authors:** M. M. H. T. van Egmond, J. P. C. Grutters, G. Hannink, N. van Heerbeek, M. M. Rovers

**Affiliations:** 1 Department of Otorhinolaryngology, Radboud Institute for Health Sciences, Radboud University Medical Center, Route 377, P.O. Box 9101, 6500 HB Nijmegen, the Netherlands; 2 Department of Operating Rooms, Radboud Institute for Health Sciences, Radboud University Medical Center, Route 715, P.O. Box 9101, 6500 HB Nijmegen, the Netherlands; 3 Department of Health Evidence, Radboud Institute for Health Sciences, Radboud University Medical Center, Route 133, P.O. Box 9101, 6500 HB Nijmegen, the Netherlands

**Keywords:** Septoplasty, Nasal obstruction, Nasal septal deviation, Quality-adjusted life year (QALY), Cost-effectiveness, Randomized controlled trial (RCT), Economic evaluation, Health policy

## Abstract

**Background:**

For years, the benefits of septoplasty have been questioned. Due to the scarce and inconclusive literature, several National Health Service (NHS) Clinical Commissioning Groups in England decided to add septal surgery to their list of restricted procedures with low clinical value. Recently, evidence was obtained that septoplasty is actually more effective than non-surgical management for nasal obstruction in adults with a deviated septum. However, the relation between costs and effects of septoplasty remains unknown.

**Methods:**

We conducted an economic evaluation alongside an open, multicenter, pragmatic randomized controlled trial in two tertiary and 16 secondary referral hospitals in the Netherlands. Adults with nasal obstruction and a deviated septum were randomized to (1) septoplasty with or without concurrent turbinate surgery or (2) non-surgical management consisting of (a combination of) medical treatment and watchful waiting. Analyses were performed on an intention-to-treat basis. Single imputation nested in the bootstrap percentile method (using 5000 bootstrap replications) was performed to assess the effect of missing data. After 12 and 24 months, we assessed the incremental costs per quality-adjusted life year (QALY) gained from a healthcare and a societal perspective.

**Results:**

A total of 203 adults were randomly assigned to septoplasty (*N* = 102) or non-surgical management (*N* = 101). After 12 months, the mean cost difference between septoplasty and non-surgical management using a healthcare or societal perspective was €1181 (95%CI €1038 to €1323) or €2192 per patient (95%CI €1714 to €2670), respectively. The mean QALY difference was 0.03 per patient (95%CI − 0.01 to 0.07). Incremental costs per QALY gained from a healthcare or societal perspective were €41,763 or €77,525, respectively. After 24 months, the mean cost difference between the two groups using a healthcare or societal perspective decreased to €936 (95%CI €719 to €1153) or €1671 per patient (95%CI €952 to €2390), respectively. The mean QALY difference increased to 0.05 per patient (95%CI − 0.03 to 0.14). Incremental costs per QALY gained from a healthcare or societal perspective became €17,374 or €31,024, respectively. Analyses of imputed data did not alter our findings.

**Conclusions:**

Depending on the selected perspective, cost-effectiveness threshold, and time horizon, septoplasty has the potential to be cost-effective. Despite considerable uncertainty, septoplasty seems to be cost-effective from a healthcare perspective, after 24 months against a threshold of €20,000 per QALY. From a societal perspective, septoplasty is not yet cost-effective after 24 months, but it comes closer to the cost-effectiveness threshold as time passes by.

**Trial registration:**

Nederlands Trial Register, NTR3868 (https://www.trialregister.nl/trial/3698). Prospectively registered on February 21, 2013.

## Background

Septoplasty (surgical correction of the deviated septum) is the most frequently performed ear, nose, and throat operation in adults, but its effectiveness has long been questioned [1]. The British National Institute for Health Research (NIHR) and professional associations of ear, nose, and throat surgeons in the UK and the Netherlands highly prioritized studies on this topic, considering it as one of the most important evidence gaps in otorhinolaryngology [2–4]. However, despite the call for further research, the literature has remained scarce and inconclusive for years [5]. As a result, several National Health Service (NHS) Clinical Commissioning Groups in England have added septal surgery to their list of restricted procedures, characterizing septoplasty as an intervention of low clinical value [6, 7]. In early 2019, the German Ear, Nose, and Throat Society released a position paper which referred to the controversy and discussed possible criteria for performing septoplasty, most notably the presence of chronic functional impairment of nasal breathing (with or without concomitant disease of the upper or lower respiratory tract) [8].

Recently, the first randomized controlled trial on the effectiveness of septoplasty showed that septal surgery is in fact superior to non-surgical management for nasal obstruction in adults with a deviated septum [9]. Both health-related quality of life and objective measurements of nasal airflow were found to improve after septoplasty. Nonetheless, previous modeling research demonstrated that septal surgery incurs significant additional costs compared to non-surgical management [10]. Considering the rising demand for care and growing strain on resources, it is important to weigh the benefits of an intervention against its costs, in order to justify its use to society [11].

Alongside the randomized controlled trial on the effectiveness of septoplasty, we collected data on healthcare and societal costs incurred by septal surgery and non-surgical management over the full 24 months of follow-up. The aim of the current study is to determine whether septoplasty is a cost-effective strategy for nasal obstruction in adults with a deviated septum, as the first step towards evidence-based policy-making in this field.

## Methods

### Design, setting, and participants

An economic evaluation was conducted alongside an open, multicenter, pragmatic randomized controlled trial in two tertiary and 16 secondary referral hospitals in the Netherlands. The study was performed in accordance with the CONSORT 2010 and CHEERS 2013 guidelines [12, 13]. The protocol was approved by the accredited medical ethics committee of the Radboud University Medical Center, Nijmegen. The study design has been reported previously [14]. In short, adults with nasal obstruction, a deviated septum, and an indication to have septoplasty performed (with or without concurrent turbinate surgery) were included in the trial between September 2013 and December 2016. We excluded patients with a history of septal surgery, patients with septal perforation, patients with untreated allergic rhinitis or allergic rhinitis unresponsive to medical treatment, patients in whom nasal obstruction was not the primary indication for septoplasty, patients in whom septoplasty was to be performed as part of a cosmetic rhinoplasty procedure, and cleft lip and/or palate patients.

After eligible patients had provided written informed consent, their demographic and disease-specific data were collected, subjective and objective outcomes were administered, and they were randomly assigned to a treatment arm.

### Randomization

An independent data manager developed a computerized minimization strategy, taking into account sex, age (< 35 years or ≥ 35 years), and severity of the deviation (mild, moderate, or severe) [15, 16]. Treatment allocation was concealed and balanced in a 1:1 ratio for the two comparators: septoplasty and non-surgical management.

### Comparators

Patients assigned to septoplasty underwent septal surgery with or without concurrent turbinate surgery as considered appropriate by their ear, nose, and throat surgeon, following regular clinical practice. The septal deviation needed to be the primary contributing factor to impaired nasal breathing. However, since turbinate enlargement often accompanies a deviated septum, additional minor turbinate surgery was allowed to prevent residual postoperative complaints of nasal obstruction [17]. The operation was scheduled within 6 to 8 weeks after the baseline visit. Patients assigned to non-surgical management underwent (a combination of) conservative strategies, which is watchful waiting and medical treatment, the latter usually consisting of local corticosteroids. Both surgical and non-surgical patients were allowed to use additional medication if needed, in line with the pragmatic nature of the trial.

### Time horizon

Follow-up visits were scheduled at 3, 6, 12, and 24 months. If patients assigned to non-surgical management underwent septoplasty within the 24 months of trial follow-up, they were classified as cross-over and monitored as planned (intention-to-treat protocol). During each follow-up visit, subjective and objective outcomes were measured and a patient-reported cost diary was collected.

### Study perspectives

An economic evaluation can be conducted from a healthcare perspective or a broader societal perspective. The healthcare perspective includes direct medical costs due to treatment and follow-up, such as medication use and healthcare contacts. The societal perspective additionally includes broader costs to society, such as direct non-medical costs (travel expenses) and indirect costs (productivity losses) [18]. The British National Institute for Health and Care Excellence (NICE) primarily focuses on costs reimbursed by the NHS and Personal Social Services (PSS) [19]. Therefore, NICE advises the use of the healthcare perspective [20]. In contrast, the Dutch guideline for economic evaluation recommends the societal perspective, taking all relevant costs into account irrespective of the payer [21]. Our analysis was performed from both a healthcare and a societal perspective.

### Measures of effectiveness

The economic evaluation was conducted using three measures of effectiveness: quality-adjusted life years (QALYs), disease-specific quality of life, and nasal airflow.

QALYs are a combination of years of life and generic health-related quality of life. Generic health-related quality of life was assessed at baseline, 3, 6, 12, and 24 months using the EuroQol five dimensions three levels (EQ-5D-3L) [22]. The EQ-5D-3L provides a measure of health state, which can be converted into a single utility score ranging from 0 (death) to 1 (full health). Utility scores were calculated using the Dutch value set [23]. Based on these utility scores, QALYs were computed using the area under the curve approach. After the first year of follow-up, QALYs were discounted at a rate of 1.5% [21].

Disease-specific quality of life was measured using the Nasal obstruction symptom evaluation (NOSE) [24, 25]. Total NOSE scores range from 0 to 100, with lower scores indicating better outcomes. To facilitate interpretation, the direction of the NOSE was inverted so that higher scores represented better results. Based on the validated minimal important change of 5.3 points on the NOSE [26], we calculated the proportion of responders (who gained 6 points or more) and non-responders (who either lost points or gained 5 points or less) for each of the two treatment strategies.

Nasal airflow in liter per minute (L/min) through both nostrils (before mucosal decongestion) was objectively assessed by means of peak nasal inspiratory flow (PNIF) [27]. The instrument used for PNIF was produced by Clement Clarke International Ltd. (Essex, UK). The average gain from baseline in nasal airflow (in percentages) was calculated for each of the two groups.

### Costs

Resource use was prospectively measured using a patient-reported cost diary, which covered the full 24 months of follow-up and was periodically collected during each study visit. The diary measured the number of resources used due to nasal complaints, such as days of medication use, healthcare contacts, kilometers traveled, hours of work lost, and hours of household work lost. Residual expenses and parking costs were obtained directly from the patient-reported cost diary. Other costs were calculated by multiplying the number of resources used with the corresponding cost price. Cost prices for healthcare contacts, kilometers traveled, hours of work lost, and hours of household work lost were based on the Dutch guideline for economic evaluation [21]. Cost prices for the medication (both prescribed and over-the-counter) were derived from the Dutch formulary [28]. The cost price of septal surgery was based on the Dutch guideline for economic evaluation and data from the Radboud University Medical Center, Nijmegen [21]. Follow-up visits for the study were excluded from the analysis. All costs were based on the 2017 price level. The average exchange rate of euro versus pound sterling and euro versus US dollar in 2017 was €1.00 = £0.88 and €1.00 = $1.13, respectively [29]. Costs occurring after the first year of follow-up were discounted at a rate of 4% [21].

### Statistical analysis

Mean differences between septoplasty and non-surgical management in costs and QALYs per patient were calculated. If septoplasty was more costly and more effective, or less costly and less effective, we computed the incremental cost-effectiveness ratio (ICER), at 12 and 24 months. This ICER represents the additional costs per QALY gained, or the costs saved per QALY lost. Following the Dutch guideline for economic evaluation, we used a cost-effectiveness threshold of €20,000 per QALY [21]. Moreover, we calculated the incremental costs per extra responder on the NOSE (gaining the validated minimal important change of 5.3 points or more) and per percentage point gain in nasal airflow as measured with PNIF, at 12 and 24 months [26]. As cost data are generally highly skewed and not normally distributed, we used BCA bootstrapping with 5000 replications to estimate 95% confidence intervals around the costs and effects [30]. Non-parametric bootstrapping with 5000 replications was used to create cost-effectiveness planes and cost-effectiveness acceptability curves. Cost-effectiveness acceptability curves were derived at 12 and 24 months, illustrating the probability of cost-effectiveness against different cost-effectiveness thresholds [31]. All analyses were performed on an intention-to-treat basis. As recommended in a recent study by Brand et al., the effect of missing data was assessed with single imputation nested in the bootstrap percentile method [32]. First, bootstrapping was used to generate 5000 incomplete datasets, and second, a single completed dataset was generated for every incomplete dataset (see Additional file 1 for a detailed description). Analyses were performed with SPSS version 22.0 (SPSS, Armonk, NY, USA), Microsoft Excel 2016 (Microsoft Corporation, Redmond, WA, USA), and R version 3.6.2 (R Project for Statistical Computing, Vienna, Austria).

## Results

### Participant characteristics

**Fig. 1:**
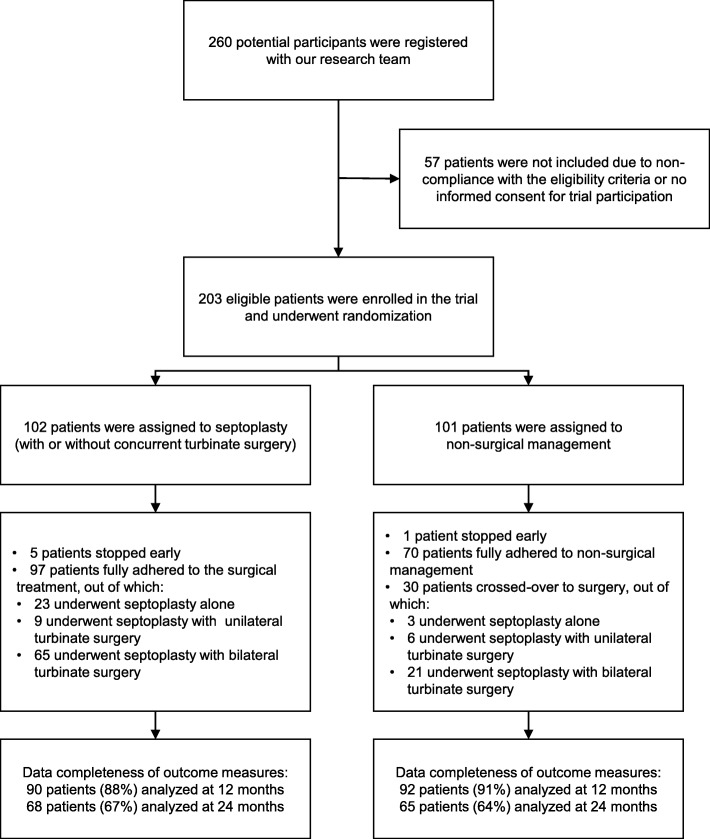
Randomization, adherence to assigned treatment, and data completeness (based on the CONSORT flow diagram)

A total of 260 potential participants were registered with our research team. As 57 patients either did not fulfill eligibility criteria or were not willing to participate, we included the remaining 203 eligible patients into the study. All included patients were randomized: 102 were assigned to septoplasty and 101 to non-surgical management. Clinical characteristics at baseline did not differ between the two groups (Table 1). The total population consisted of more males than females (*N* = 138, 68%), the mean age was 38 years (SD 15), and the median duration of nasal obstruction before trial entry was 7 years (IQR 12). Analyses at 12 months were conducted in 90 patients (88%) assigned to septoplasty and 92 patients (91%) assigned to non-surgical management, and at 24 months, in 68 patients (67%) assigned to septoplasty and 65 patients (64%) assigned to non-surgical management (Fig. 1).

**Table 1:** Baseline characteristics of the trial population

Characteristic	Septoplasty (with or without concurrent turbinate surgery), *N* = 102	Non-surgical management, *N* = 101
Age, years		
Mean ± SD	39 ± 14	37 ± 15
Range	18 to 67	18 to 70
Male sex, no. (%)	73 (72)	66 (65)
Body mass index		
Mean ± SD	25.0 ± 4.5	25.6 ± 3.8
Range	16.7 to 39.9	17.5 to 38.7
Duration of nasal obstruction, years		
Median	6	8
IQR	10	11
Nasal obstruction bilateral, no. (%)	64 (63)	61 (60)
Previous treatment for nasal obstruction, no. (%)	81 (79)	87 (86)
Local corticosteroids, no. (%)	73 (72)	73 (72)
Nasal decongestants, no. (%)	39 (38)	42 (42)
Turbinate surgery, no. (%)	2 (2)	3 (3)
History of nasal trauma, no. (%)	42 (41)	42 (42)
Smoking status		
Current smoker, no. (%)	26 (25)	34 (34)
History of smoking, no. (%)	34 (33)	30 (30)
Cocaine abuse, no. (%)	7 (7)	11 (11)
Snoring^†^, no. (%)	50 (49)	44 (44)
Allergic rhinitis^†^, no. (%)	32 (31)	30 (30)
Asthma^†^, no. (%)	24 (24)	18 (18)
Turbinate hypertrophy, no. (%)	46 (45)	47 (47)
Unilateral, no. (%)	29 (28)	24 (24)
Bilateral, no. (%)	16 (16)	18 (18)
Septal deviation bilateral, no. (%)	25 (25)	23 (23)
Septal deviation severity^‡^		
Mild, no. (%)	32 (31)	27 (27)
Moderate, no. (%)	66 (65)	67 (66)
Severe, no. (%)	37 (36)	32 (32)
Measures of effectiveness		
EQ-5D-3L utility score, mean ± SD	0.83 ± 0.19	0.82 ± 0.19
EQ-5D-3L VAS, mean ± SD	72.1 ± 16.1	74.1 ± 17.0
NOSE, mean ± SD	32.8 ± 18.1	34.4 ± 19.3
PNIF, mean ± SD^§^	91.6 ± 39.0	87.1 ± 40.5

^†^Self-reported; no diagnostics performed as part of the trial

^‡^The deviation was classified by the ear, nose, and throat surgeon as mild if it obstructed less than half of the nasal passage, as moderate if it obstructed half or more than half of the nasal passage, and as severe if the deviation was in contact with the lateral nasal wall

^§^Presented values are before the decongestion of the nasal mucosa

### Costs

An overview of the resources used and the corresponding cost prices is provided in Table 2. The mean component and total costs per patient from a healthcare or societal perspective after 12 and 24 months for each of the two treatment strategies are shown in Table 3.

**Table 2:** Resources used with their corresponding cost price for 2017. The average exchange rate of euro versus pound sterling and euro versus US dollar in 2017 was €1.00 = £0.88 and €1.00 = $1.13, respectively [29]

Resource	Cost price in €	Source
Medication use (per day)		
Local corticosteroids	0.38	Formulary
Oral corticosteroids	0.14	Formulary
Decongestants	0.33	Formulary
Analgesics	0.85	Formulary
Antibiotics	1.87	Formulary
Antihistamines	0.23	Formulary
Saline solution	0.07	Formulary
Nasal ointment	0.67	Formulary
Healthcare contacts		
General practitioner visit during office hours	34.00	Guideline
General practitioner visit after office hours	109.11	Guideline
General practitioner telephone consultation during office hours	17.00	Guideline
General practitioner telephone consultation after office hours	25.00	Guideline
Medical specialist visit during office hours	93.00	Guideline
Medical specialist visit after office hours	265.00	Guideline
Medical specialist telephone consultation during office hours	19.79	Guideline
Medical specialist telephone consultation after office hours	29.03	Guideline
Medical assistant/therapist visit during office hours	33.00	Guideline
Surgery		
Septoplasty (with or without concurrent turbinate surgery)	1241	Guideline and Radboudumc
Travel expenses		
Taxi kilometer fare	2.72	Guideline
Taxi start rate	3.02	Guideline
Car/motor/public transport kilometer fare	0.19	Guideline
Parking	NA	Cost diary
Productivity losses		
Hour of work lost (due to healthcare visits)	34.36	Guideline
Hour of household work lost (due to healthcare visits)	14.32	Guideline
Hour of work or household work lost (due to illness or postoperative recovery)	32.64	Guideline
Residual expenses		
Paid help	NA	Cost diary
Others	NA	Cost diary

*Cost diary* patient-reported cost diary, *Formulary* Dutch formulary [28], *Guideline* Dutch guideline for economic evaluation [21], *NA* not applicable, *Radboudumc* Radboud University Medical Center, Nijmegen

**Table 3:** Mean component costs and mean total healthcare and societal costs per patient for septoplasty and non-surgical management after 12 and 24 months. The average exchange rate of euro versus pound sterling and euro versus US dollar in 2017 was €1.00 = £0.88 and €1.00 = $1.13, respectively [29]

Resource	Mean costs in € (95%CI)			
	Septoplasty, 0–12 months (*N* = 90)	Non-surgical management, 0–12 months (*N* = 92)	Septoplasty, 0–24 months (*N* = 68)	Non-surgical management, 0–24 months (*N* = 65)
Medication use	41 (31 to 53)	103 (87 to 119)	56 (38 to 78)	150 (123 to 179)
Healthcare contacts	387 (355 to 423)	117 (82 to 155)	366 (334 to 400)	219 (143 to 313)
Surgery	1241 (1241 to 1241)	270 (172 to 379)	1241 (1241 to 1241)	358 (227 to 492)
Total healthcare costs	1670 (1634 to 1710)	489 (359 to 622)	1663 (1623 to 1706)	727 (533 to 941)
Travel expenses	37 (31 to 44)	9 (5 to 13)	41 (34 to 50)	15 (7 to 24)
Productivity losses	1680 (1429 to 1947)	714 (475 to 1002)	1707 (1399 to 2019)	999 (648 to 1416)
Residual expenses	18 (3 to 36)	1 (0 to 2)	12 (3 to 26)	11 (2 to 24)
Total societal costs	3404 (3108 to 3702)	1212 (881 to 1575)	3423 (3088 to 3767)	1752 (1234 to 2393)

#### Healthcare perspective

After 12 months, the mean healthcare costs per patient assigned to septoplasty and non-surgical management were €1670 (95% CI €1634 to €1710) and €489 (95% CI €359 to €622), respectively. The mean cost difference between the two groups was €1181 (95% CI €1038 to €1323).

After 24 months, the mean healthcare costs per surgical and non-surgical patient were €1663 (95% CI €1623 to €1706) and €727 (95% CI €533 to €941), respectively. The mean cost difference between the two groups decreased to €936 (95% CI €719 to €1153).

#### Societal perspective

After 12 months, the mean societal costs per patient assigned to septoplasty and non-surgical management were €3404 (95% CI €3108 to €3702) and €1212 (95% CI €881 to €1575), respectively. The mean cost difference between the two groups was €2192 (95%CI €1714 to €2670).

After 24 months, the mean societal costs per surgical and non-surgical patient became €3423 (95%CI €3088 to €3767) and €1752 (95%CI €1234 to €2393), respectively. The mean cost difference between the two groups decreased to €1671 (95%CI €952 to €2390).

### Economic evaluation

#### Costs per QALY

After 12 months, patients assigned to septoplasty and non-surgical management had on average 0.88 QALYs (95%CI 0.85 to 0.91) and 0.85 QALYs (95%CI 0.83 to 0.88), respectively. The mean QALY difference between the two groups was 0.03 (95%CI − 0.01 to 0.07). Incremental costs per QALY gained from septoplasty were €41,763 from a healthcare perspective and €77,525 from a societal perspective.

After 24 months, surgical and non-surgical patients had on average 1.78 QALYs (95%CI 1.71 to 1.84) and 1.73 QALYs (95%CI 1.67 to 1.78), respectively. The mean QALY difference between the two treatment strategies increased to 0.05 (95%CI − 0.03 to 0.14). Incremental costs per QALY gained from septoplasty became €17,343 from a healthcare perspective and €31,024 from a societal perspective.

#### NOSE

After 12 months, 74 out of 90 surgical patients (82%) and 49 out of 92 non-surgical patients (53%) could be classified as responders on the NOSE, gaining the validated minimal important change of 5.3 points or more [26]. The mean difference between the two groups in percentage point response was 29 (95%CI 16 to 42). Based on a mean difference in societal costs between the two groups of €2192 (95%CI €1714 to €2670), incremental costs per extra responder on the NOSE were €7568.

After 24 months, 53 out of 68 surgical patients (78%) and 30 out of 65 non-surgical patients (46%) were responders, gaining the validated minimal important change of 5.3 points or more [26]. The mean difference between the two groups in percentage point response was 32 (95% CI 16 to 48). Given a mean difference in societal costs between the two groups of €1671 (95%CI €952 to €2390), incremental costs per extra responder on the NOSE were €5256.

#### PNIF

After 12 months, the mean gain from baseline in nasal airflow as measured with PNIF in surgical and non-surgical patients was 45% (95% CI 34 to 56%) and 24% (95% CI 11 to 37%), respectively. The mean difference between the two groups in percentage point gain in nasal airflow was 21 (95% CI 4 to 38). Based on a mean difference in societal costs between the two groups of €2384 (95% CI €1873 to €2895), incremental costs per percentage point gain in nasal airflow were €113.

After 24 months, the mean gain from baseline in nasal airflow as measured with PNIF in surgical and non-surgical patients was 40% (95% CI 27 to 55%) and 28% (95% CI 13 to 43%), respectively. The mean difference between the two groups in percentage point gain in nasal airflow was 13 (95%CI − 9 to 35). Given a mean difference in societal costs between the two groups of €1790 (95%CI €777 to €2803), incremental costs per percentage point gain in nasal airflow were €139.

### Uncertainty

**Fig. 2:**
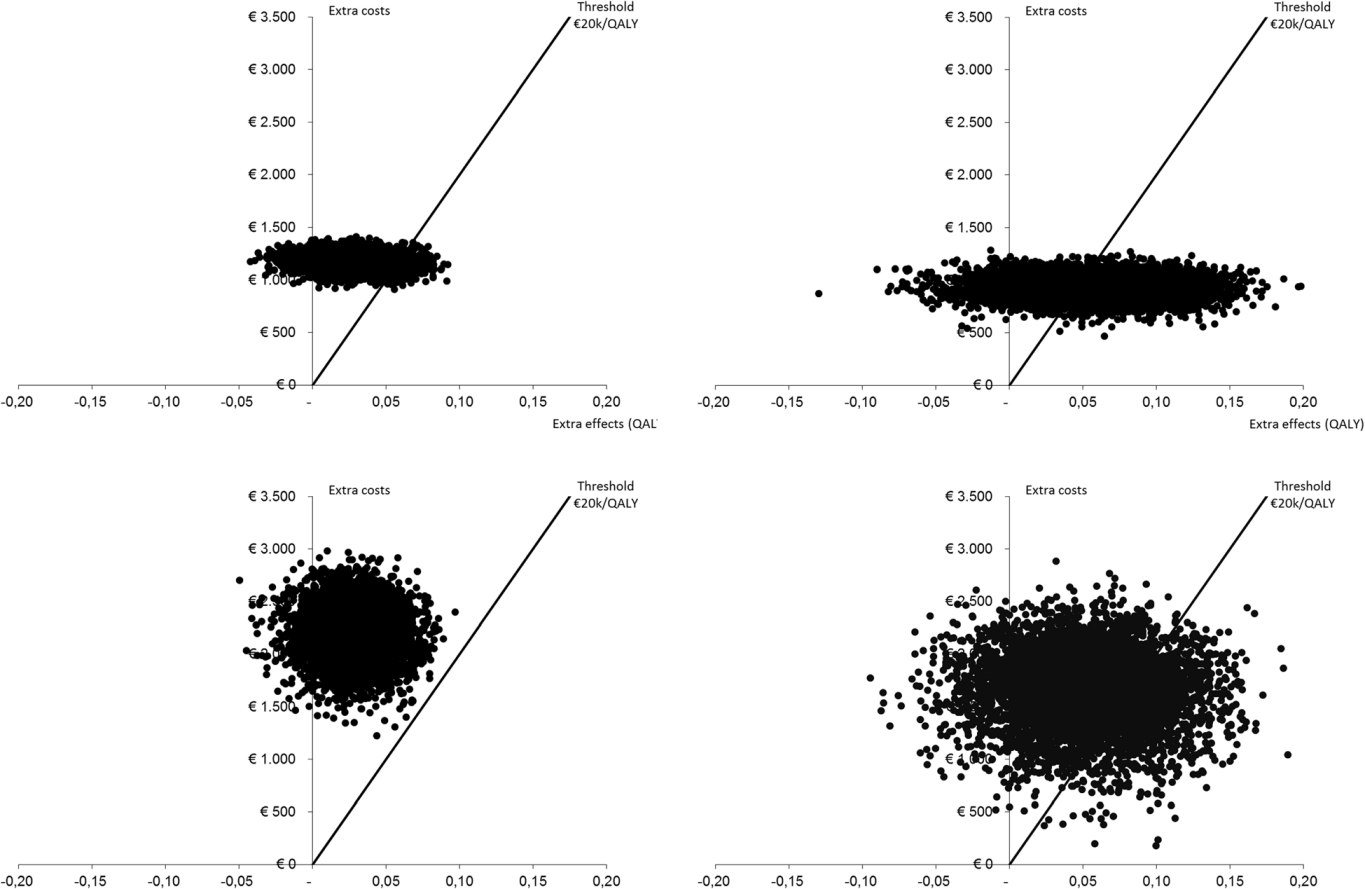
**a** Cost-effectiveness plane. Graphical overview of the incremental costs in euro (*y*-axis) per QALY gained (*x*-axis) for each of the bootstrap replications from a healthcare perspective after 12 months. **b** Cost-effectiveness plane. Graphical overview of the incremental costs in euro (*y*-axis) per QALY gained (*x*-axis) for each of the bootstrap replications from a healthcare perspective after 24 months. **c** Cost-effectiveness plane. Graphical overview of the incremental costs in euro (*y*-axis) per QALY gained (*x*-axis) for each of the bootstrap replications from a societal perspective after 12 months. **d** Cost-effectiveness plane. Graphical overview of incremental costs in euro (*y*-axis) per QALY gained (*x*-axis) for each of the bootstrap replications from a societal perspective after 24 months

Cost-effectiveness planes, providing a graphical overview of incremental costs and QALYs after 12 and 24 months for each of the bootstrap replications, are shown in Fig. 2a and b for the healthcare perspective and Fig. 2c and d for the societal perspective.

**Fig. 3:**
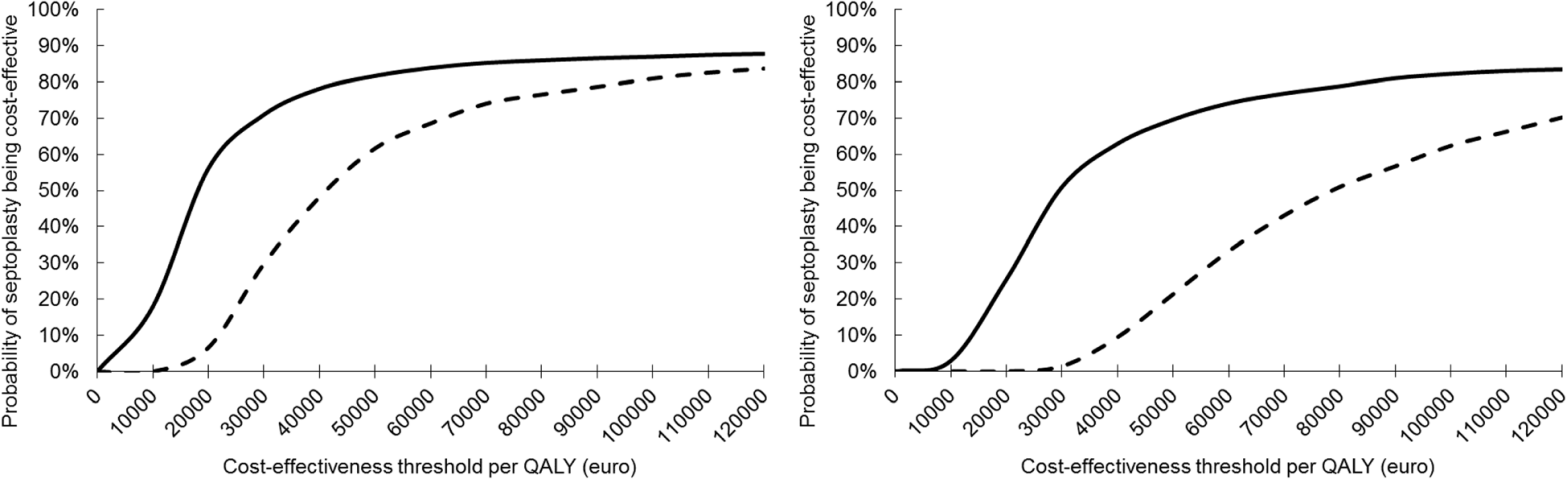
**a** Acceptability curve illustrating the probability that septoplasty is cost-effective from a healthcare perspective (*y*-axis) at different cost-effectiveness thresholds (*x*-axis) after 12 months (dashed line) and 24 months (solid line). Against a threshold of €20,000 per QALY, septoplasty was cost-effective in 7% of the replications after 12 months and in 56% of the replications after 24 months. **b** Acceptability curve illustrating the probability that septoplasty is cost-effective from a societal perspective (*y*-axis) at different cost-effectiveness thresholds (*x*-axis) after 12 months (dashed line) and 24 months (solid line). Against a threshold of €20,000 per QALY, septoplasty was cost-effective in 0% of the replications after 12 months and in 25% of the replications after 24 months

Acceptability curves, illustrating the probability that septoplasty is cost-effective after 12 and 24 months against different cost-effectiveness thresholds, are provided in Fig. 3a for the healthcare perspective and Fig. 3b for the societal perspective. From a healthcare perspective, septoplasty was cost-effective in 7% of the replications after 12 months and in 56% of the replications after 24 months, and from a societal perspective, septoplasty was cost-effective in 0% of the replications after 12 months and in 25% of the replications after 24 months, all against a cost-effectiveness threshold of €20,000 per QALY. In line with the base-case estimates, the majority of bootstrap replications thus indicate that septoplasty is cost-effective from a healthcare perspective after 24 months. Furthermore, while septoplasty is not yet cost-effective from a societal perspective after 24 months, it progresses towards the cost-effectiveness threshold as time passes by.

### Imputation of missing data

Table S1, Figures S1a-S1d, and Figures S2a-S2b in Additional file 1 provide an overview of the complete case analyses and single imputation nested in the bootstrap percentile method (using 5000 bootstrap replications). Generally, the results of both methods were comparable. The difference between septoplasty and non-surgical management in healthcare costs at 12 and 24 months and societal costs at 12 months was slightly smaller with single imputation nested in the bootstrap percentile method than with complete case analyses, thereby increasing the cost-effectiveness of septal surgery. The only exception was societal costs at 24 months. However, this did not affect the conclusion, as the complete case analyses too demonstrated that septoplasty was not yet cost-effective from a societal perspective after two years.

## Discussion

### Principal findings

This economic evaluation, performed alongside a randomized controlled trial, showed that septoplasty is both more effective and more costly than non-surgical management for nasal obstruction in adults with a deviated septum. However, surgical costs are predominantly incurred in the first year, whereas costs of non-surgical management gradually accumulate as the treatment continues. Accordingly, the cost difference between surgical and non-surgical treatment was found to decrease over time, while the difference in health-related quality of life between the two groups persisted. Incremental costs per QALY gained after 12 months were €41,763 from a healthcare perspective and €77,525 from a societal perspective. After 24 months, incremental costs per QALY gained became €17,374 from a healthcare perspective and €31,024 from a societal perspective. Given a cost-effectiveness threshold of €20,000 per QALY, septoplasty is cost-effective from a healthcare perspective. While septoplasty is not yet cost-effective from a societal perspective after 2 years, it comes closer to the cost-effectiveness threshold as time passes by.

### Comparison with the literature

Randomized controlled trials or non-randomized comparative studies on the (cost-) effectiveness of septoplasty have not been published before [5]. Previous modeling research based on publicly available data sources demonstrated that septoplasty incurs significant additional costs compared to non-surgical management, especially considering the productivity losses due to postoperative recovery [10]. However, as effectiveness data were lacking, the relation between costs and effects of septal surgery remained unclear.

### Strengths and weaknesses

The main strength of our study is that this is the first trial-based economic evaluation of septoplasty. Data on costs and effects were prospectively and simultaneously collected in an open, multicenter, pragmatic randomized controlled trial. The economic evaluation was conducted from a healthcare and societal perspective and was performed and reported in accordance with the CONSORT 2010 and CHEERS 2013 guidelines [12, 13].

Some of our findings deserve further attention. First, the study’s follow-up was limited to 24 months. Because surgery is relatively costly in the first year but saves expenses in later years, the probability of septoplasty being cost-effective increases as time passes. From a healthcare perspective, septal surgery is already cost-effective after 24 months. From a societal perspective, septoplasty needs longer to compensate for its extra costs but will most likely become cost-effective over the course of the third or fourth postoperative year. This scenario holds even if the QALY difference stabilizes after the second year, provided that the cost difference continues to decrease due to ongoing expenses for non-surgical treatment.

Second, generic health-related quality of life was measured with the EQ-5D-3L, but this instrument suffers from limited sensitivity and ceiling effects [9, 33]. This may have resulted in an underestimation of the QALY difference between surgical and non-surgical patients. Even with the EQ-5D-3L, however, septoplasty was found to be (potentially) cost-effective.

Third, our randomized controlled trial included four other measures of effectiveness that were not taken into account in the economic evaluation [14]. This is because those instruments were less suited for the purpose of an economic evaluation: the EQ-5D-3L was the only instrument that could be used for QALY calculations; the NOSE was the only validated instrument for which a minimal important change was established; and PNIF was the objective instrument that appeared to assess nasal patency best [9, 22, 26]. All the measures of effectiveness included in our economic evaluation seem to indicate that septoplasty provides value for money.

Fourth, while surgical patients were regularly monitored by their operating ear, nose, and throat surgeon regardless of the study visits, non-surgical patients may have used the study visits as a replacement for regular outpatient consultation. This could be disadvantageous for the cost-effectiveness of septoplasty, as study visits were excluded from the analysis.

Fifth, most patients assigned to surgery underwent septoplasty with concurrent turbinate surgery, rather than septoplasty alone. Concurrent turbinate surgery was allowed, given the pragmatic nature of the trial. Stratified analysis performed as part of our effectiveness study did not point towards the potential modification of the effect of septoplasty [9]. From an economic perspective, the impact is limited as well: the costs of septoplasty alone and septoplasty with concurrent turbinate surgery are comparable, as the additional time investment is negligible and the materials required for turbinate surgery are routinely present during septoplasty. Given the limited number of patients who underwent septoplasty alone, however, we feel that our trial does not allow for firm conclusions about the (cost-)effectiveness of concurrent turbinate surgery.

Sixth, a total of 21 (10%) patients at 12 months and 70 (35%) patients at 24 months had (some) missing data. The majority of the missing values at 24 months were caused by the fixed stop date of the trial and were thus considered to be missing completely at random. In this case, complete case analysis provides valid and unbiased results reflecting the true estimates. Still, the effect of missing data was examined with single imputation nested in the bootstrap percentile method (using 5000 bootstrap replications), as recommended by Brand et al. [32]. These analyses of imputed data did not alter our findings.

Seventh, blinding of participants was impossible, as medical ethics committees in the Netherlands consider it unethical to perform sham surgery in the control group. However, we used both subjective and objective outcome measures, which showed similar results.

In short, the conclusion that septal surgery has the potential to compensate its extra costs appears to be robust. Some of the issues discussed above may have been unfavorable to septoplasty, but it was still cost-effective from a healthcare perspective after 24 months.

### Clinical implications

The effectiveness of septoplasty has long been questioned. Over the past decades, doubts surrounding its benefits have led to an increasing strain on septal surgery, as demonstrated by the decision of several NHS Clinical Commissioning Groups in England to add septoplasty to their list of restricted procedures with low clinical value. However, this study shows that septoplasty is not only more effective than non-surgical management for nasal obstruction in adults with a deviated septum, but also potentially cost-effective, depending on the selected perspective, cost-effectiveness threshold, and time horizon. From a healthcare perspective, septoplasty is already cost-effective after 24 months against the Dutch threshold of €20,000 per QALY and the British threshold of £20,000 (€22,814) per QALY. From a societal perspective, septoplasty’s incremental costs per QALY gained are expected to fall below the Dutch cost-effectiveness threshold over the course of the third or fourth postoperative years. With this study, we aim to inspire a debate between all stakeholders (including patients, healthcare providers, health insurance companies, and policy-makers) as the first step towards evidence-based policy development on septal surgery in this target population, taking the above findings into account. One of the issues that needs to be addressed is the indication for septoplasty. In light of our findings, existing restrictions placed on the use and reimbursement of septoplasty may seem inopportune. At the same time, we must remain cautious of extending the indication beyond the current evidence base.

## Conclusions

Septoplasty is both more effective and more costly than non-surgical management for nasal obstruction in adults with a deviated septum. Depending on the selected perspective, cost-effectiveness threshold, and time horizon, septoplasty has the potential to be cost-effective. Despite considerable uncertainty, septoplasty seems cost-effective from a healthcare perspective, after 24 months against a threshold of €20,000 per QALY. From a societal perspective, septoplasty is not yet cost-effective after 24 months, but it comes closer to the cost-effectiveness threshold as time passes by.

## Supplementary information

**Additional file 1.** Missing data and single imputation nested in the bootstrap percentile method.

## Data Availability

De-identified participant data will be made available on reasonable request to researchers whose proposed use of the data has been approved by the principal investigators (NvH and MR), with a signed data access agreement and only for purposes specified in the approved research proposal. Requests for data must be sent to Niels.vanHeerbeek@radboudumc.nl. The study protocol is publicly available online.

## Abbreviations

EQ-5D-3L: EuroQol five dimensions three levels
ICER: Incremental cost-effectiveness ratio
NHS: National Health Service
NICE: British National Institute for Health and Care Excellence
NIHR: British National Institute for Health Research
NOSE: Nasal obstruction symptom evaluation
PNIF: Peak nasal inspiratory flow
PSS: Personal social services
QALY: Quality-adjusted life year
RCT: Randomized controlled trial
