# Supplementary material for: Septoplasty versus non-surgical management for nasal obstruction in adults with a deviated septum: economic evaluation alongside a randomized controlled trial
Source: BMC Med. 2020 May 1;18:101. doi: 10.1186/s12916-020-01562-5 (PMC7193380; doi:10.1186/s12916-020-01562-5)
Supplement: Supplementary file 1 — Additional file 1. Missing data and single imputation nested in the bootstrap percentile method. [file 12916_2020_1562_MOESM1_ESM.zip › SUPPLEMENTARY APPENDIX.docx]

**SUPPLEMENTARY APPENDIX**

**Missing data and single imputation nested in the bootstrap percentile method**

In this study, 32.5% of eligible participants had at least one missing value for the set of variables considered relevant for the subsequent analyses of incremental costs per quality-adjusted life year (QALY) gained from a healthcare and a societal perspective after 12 and 24 months. At 12 months, fractions of missing values in individual variables were between 6.1% and 7.6%. At 24 months, fractions of missing values in individual variables were between 31.0% and 32.5%. The distribution of missing data was balanced in both arms.

Missing and skewed data are common to economic evaluations and are often dealt with through multiple imputation and bootstrapping, respectively. A recent study by Brand et al explores how these methods can be combined.^(1)^ Brand et al assessed the statistical validity and efficiency of ten candidate methods using simulation, and applied these methods to a clinical dataset. Single imputation nested in the bootstrap percentile method emerged as the method with the most favorable statistical properties for handling missing and skewed data.^(1)^

We used single imputation nested in the bootstrap percentile method (using 5,000 bootstrap replications) to predict the outcome in participants with missing outcome data. First, bootstrapping was used to generate 5,000 incomplete datasets. Second, a single completed dataset was generated for every incomplete dataset. With single imputation nested in the bootstrap percentile method, no pooling over the imputations is needed. Single imputation does not only impute the expected value of missing data, but also adds ‘noise’ to reflect the uncertainty of the imputation (by using a single call to the ‘mice’-procedure per bootstrap resample). This analysis was performed using the ‘mice’ (version 3.7.0) and 'boot' (version 1.3-24) package in R version 3.6.2 (R Project for Statistical Computing, Vienna, Austria).

In detail, for the single imputations we used multivariate imputation by chained equations. This method assumes that data are missing at random (MAR), meaning that any systematic differences between the observed and missing values can be explained by differences in the observed data. To satisfy the assumption of data MAR, it is recommended to perform a so-called inclusive analysis strategy, incorporating a number of auxiliary variables into the analysis model or into the imputation process.^(2)^ We performed an inclusive analysis strategy and additionally used conditional histograms to inspect if the missingness of the outcome variables depended on the other variables (e.g., right-tailed or left-tailed MAR missingness). We used information about the treatment allocation and all available values of baseline variables and outcomes to predict missing values using predictive mean matching. Convergence of the chained equation procedure was visually evaluated from trace plots of the mean and standard deviation of the imputed data against iteration number.

Table S1 shows the analyses of complete cases compared to single imputation nested in the bootstrap percentile method (using 5,000 bootstrap replications).

Figures S1a-S1d and Figures S2a-S2b show cost-effectiveness planes and acceptability curves for both complete cases and single imputation nested in the bootstrap percentile method (using 5,000 bootstrap replications).

**TABLES SUPPLEMENTARY APPENDIX**

**Table S1**. Complete case analyses versus single imputation nested in the bootstrap percentile method (using 5,000 bootstrap replications).

| **Parameter** | **Complete cases**  Point estimate (95% CI) | **Single imputation nested in the bootstrap percentile method**  Point estimate (95% CI) |
| --- | --- | --- |
| Cost difference from a healthcare perspective at 12 months (in €) | 1,181  (1,038 to 1,323) | 1,134  (993 to 1,271) |
| Cost difference from a healthcare perspective at 24 months (in €) | 936  (719 to 1,153) | 728  (442 to 1,050) |
| Cost difference from a societal perspective at 12 months (in €) | 2,192  (1,714 to 2,670) | 2,046  (1,575 to 2,493) |
| Cost difference from a societal perspective at 24 months (in €) | 1,671  (952 to 2,390) | 1,839  (108 to 2,115) |
| QALY difference at 12 months | 0.03  (-0.01 to 0.07) | 0.03  (-0.01 to 0.07) |
| QALY difference at 24 months | 0.05  (-0.03 to 0.14) | 0.04  (-0.02 to 0.13) |

**FIGURES SUPPLEMENTARY APPENDIX**


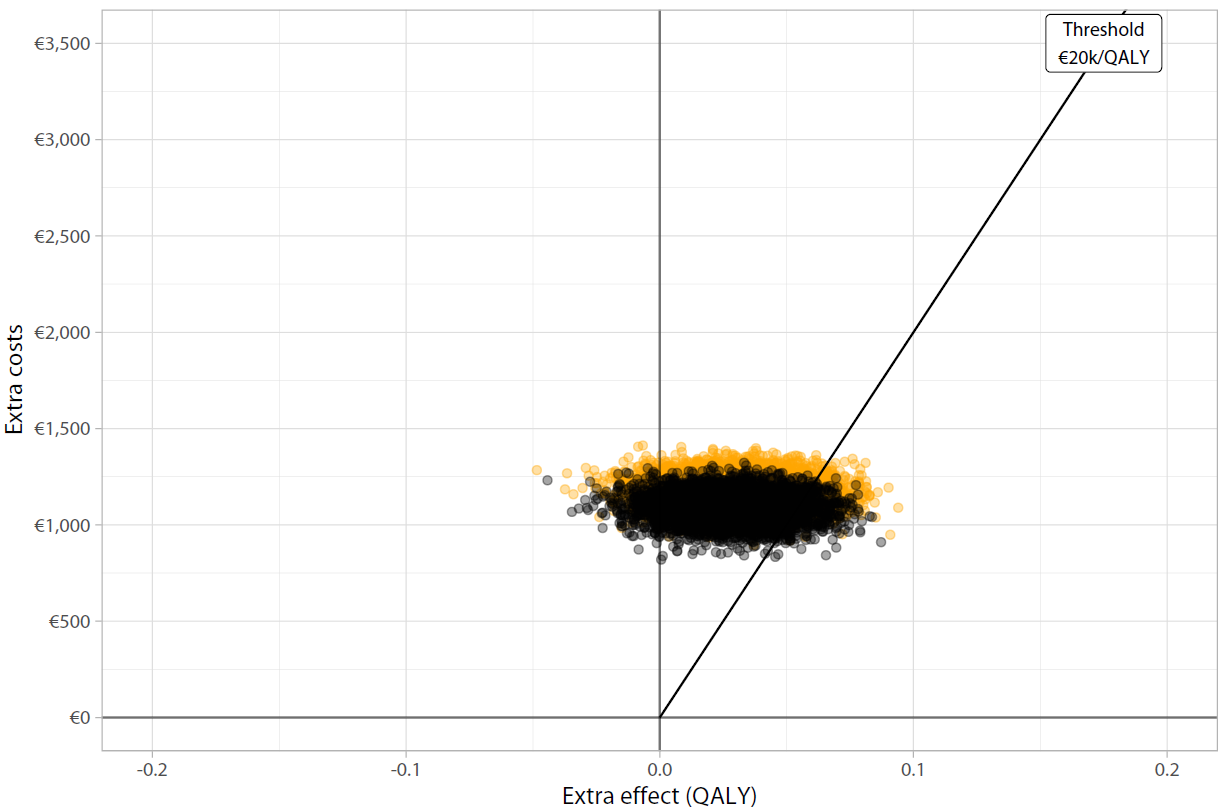
**Figure S1a.** Cost-effectiveness plane: graphical overview of incremental costs in euro (y-axis) per QALY gained (x-axis) for each of the bootstrap replications from a healthcare perspective after 12 months, for both complete cases (orange) and single imputation nested in the bootstrap percentile method using 5,000 bootstrap replications (black).


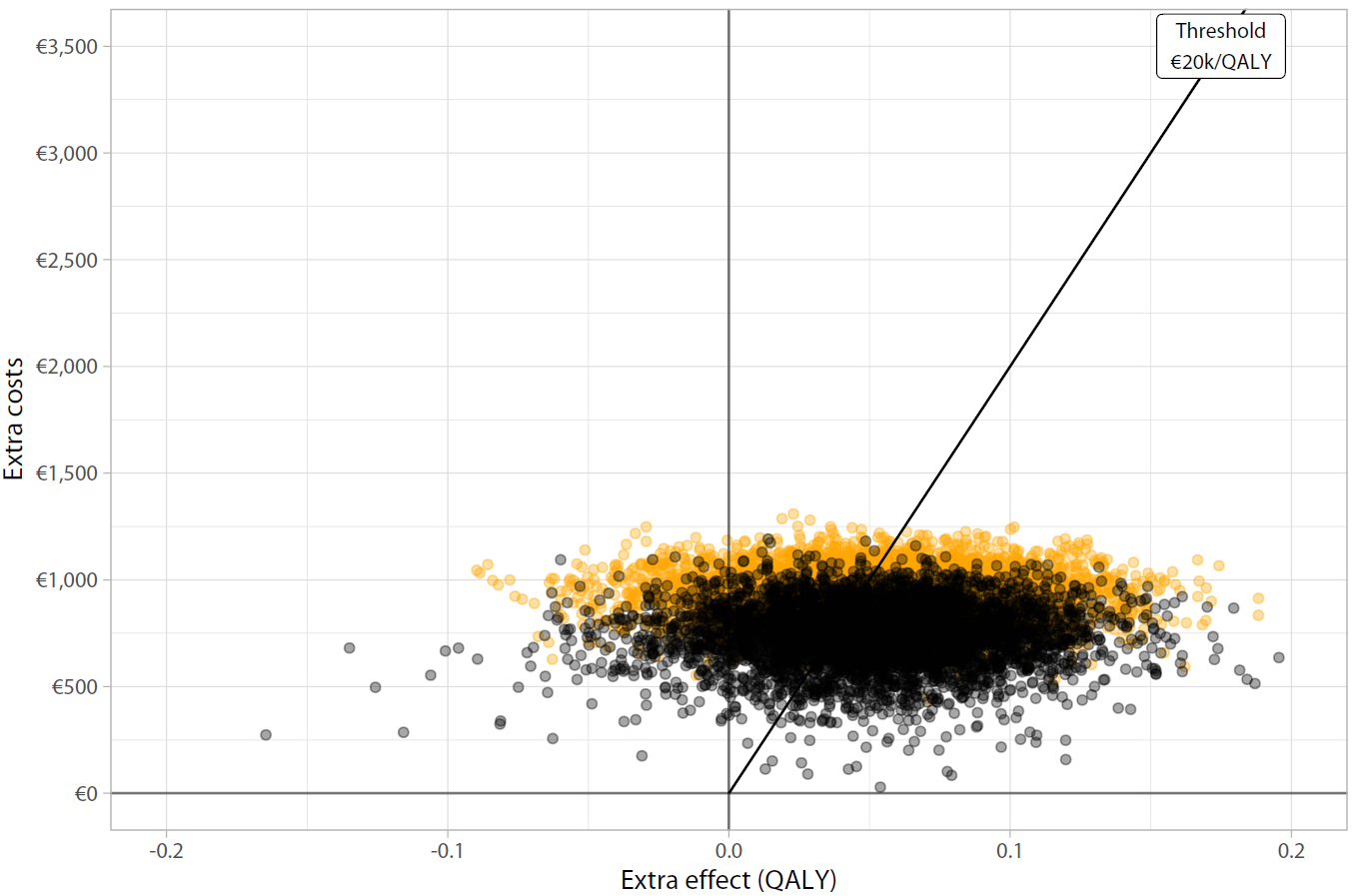
**Figure S1b.** Cost-effectiveness plane: graphical overview of incremental costs in euro (y-axis) per QALY gained (x-axis) for each of the bootstrap replications from a healthcare perspective after 24 months, for both complete cases (orange) and single imputation nested in the bootstrap percentile method using 5,000 bootstrap replications (black).


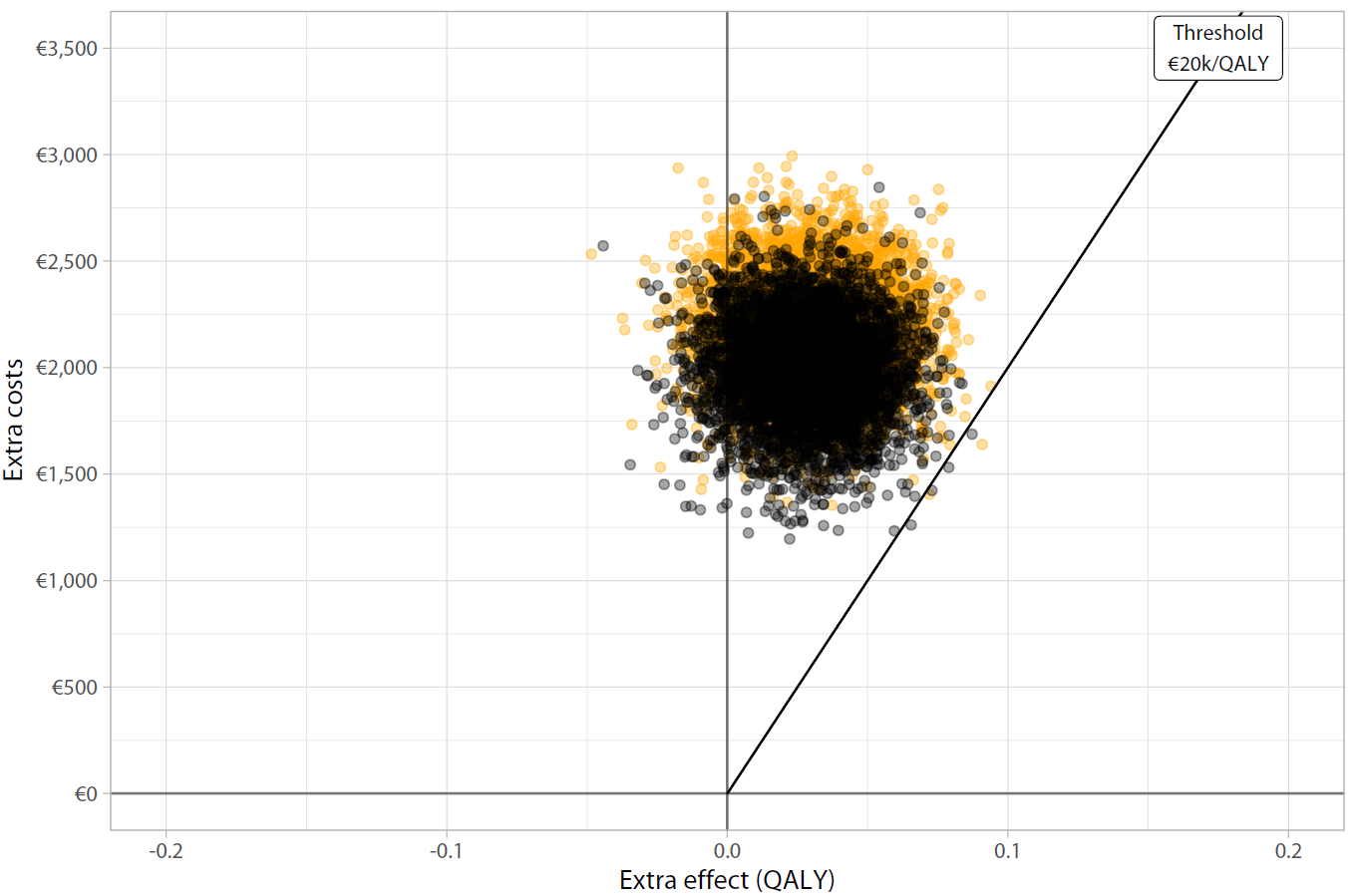
**Figure S1c.** Cost-effectiveness plane: graphical overview of incremental costs in euro (y-axis) per QALY gained (x-axis) for each of the bootstrap replications from a societal perspective after 12 months, for both complete cases (orange) and single imputation nested in the bootstrap percentile method using 5,000 bootstrap replications (black).


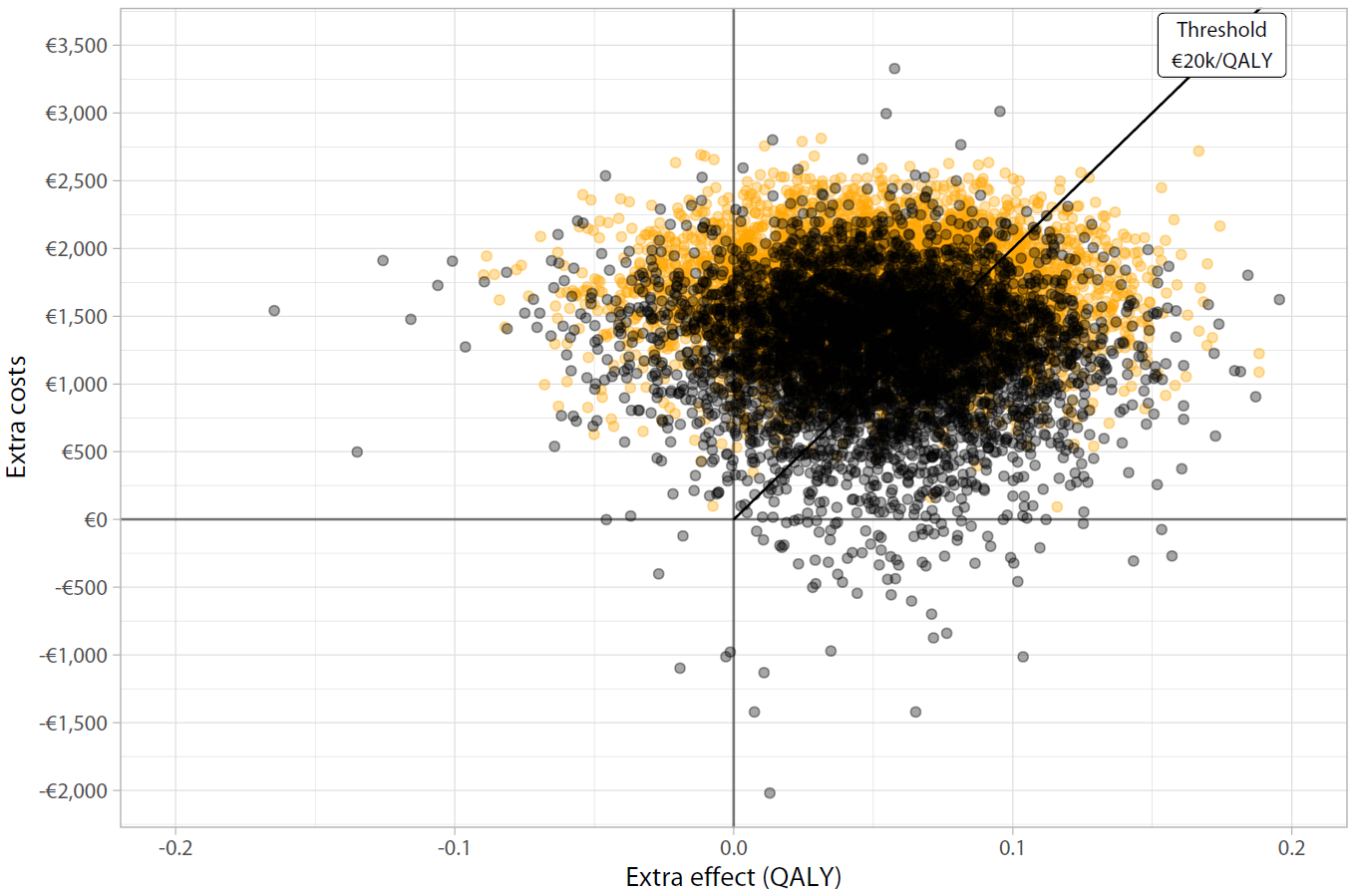
**Figure S1d.** Cost-effectiveness plane: graphical overview of incremental costs in euro (y-axis) per QALY gained (x-axis) for each of the bootstrap replications from a societal perspective after 24 months, for both complete cases (orange) and single imputation nested in the bootstrap percentile method using 5,000 bootstrap replications (black).


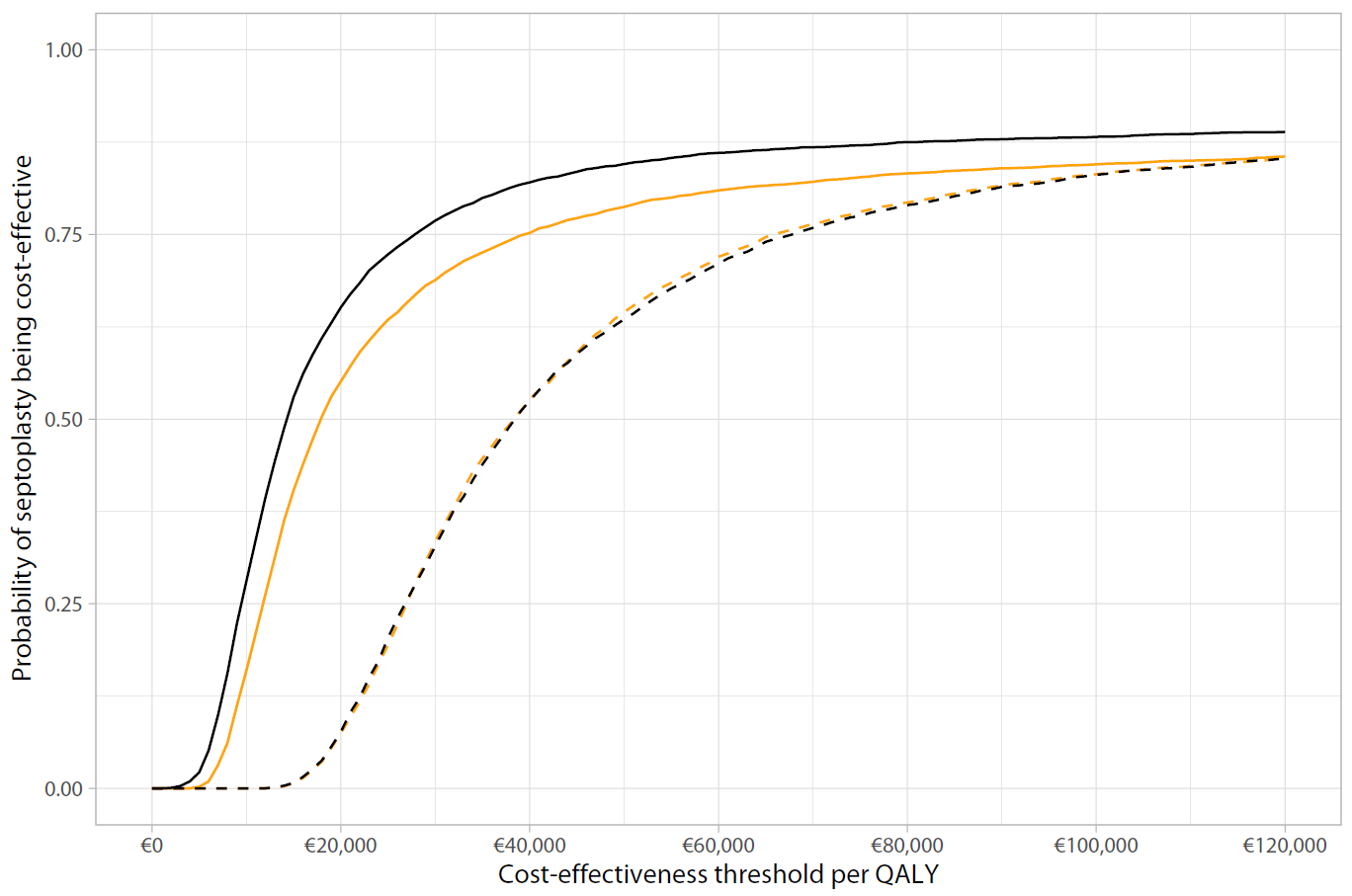
**Figure S2a.** Acceptability curve illustrating the probability that septoplasty is cost-effective from a healthcare perspective (y-axis) at different cost-effectiveness thresholds (x-axis) after 12 months (dashed line) and 24 months (solid line), for both complete cases (orange) and single imputation nested in the bootstrap percentile method using 5,000 bootstrap replications (black).


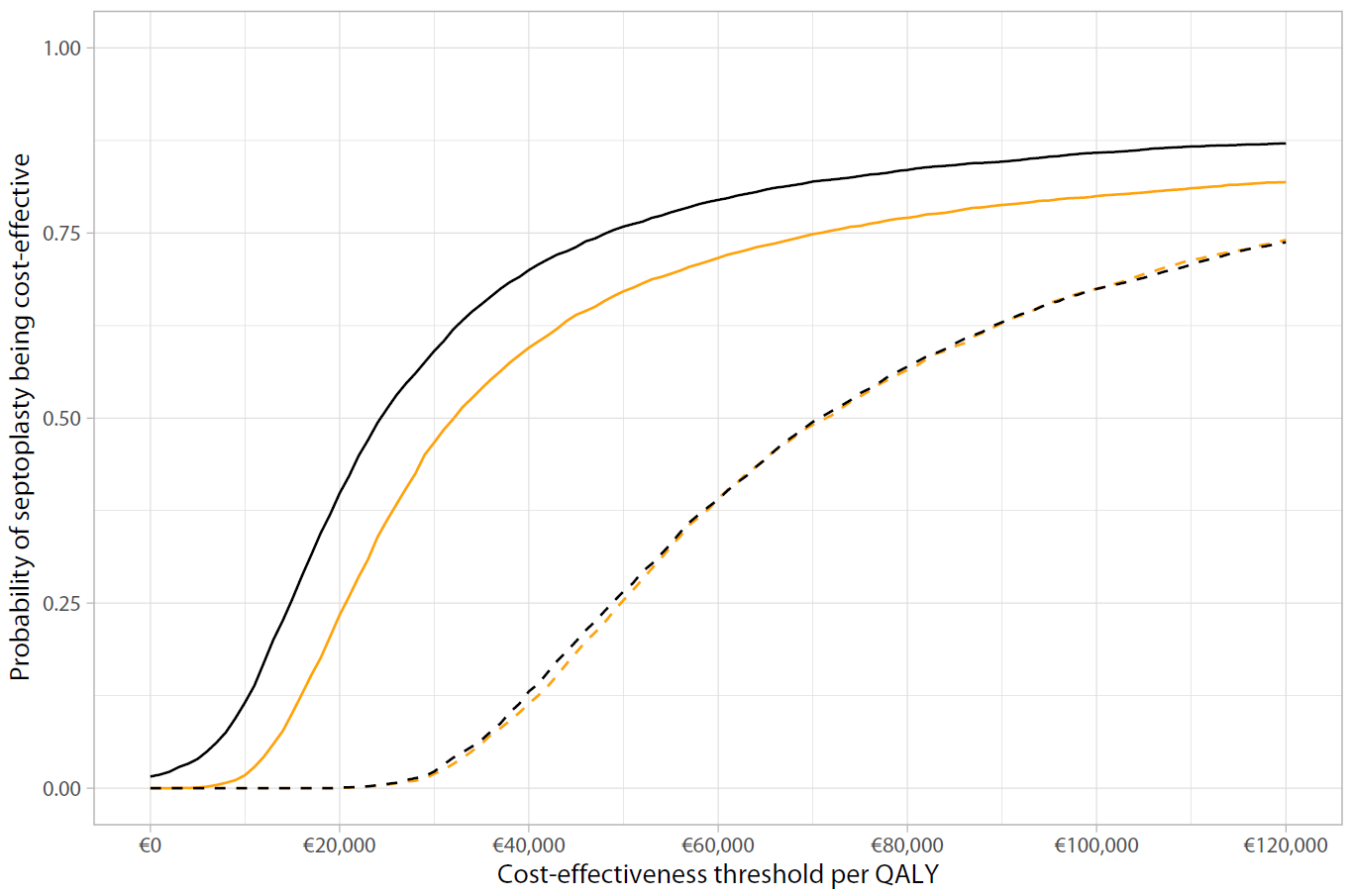
**Figure S2b.** Acceptability curve illustrating the probability that septoplasty is cost-effective from a societal perspective (y-axis) at different cost-effectiveness thresholds (x-axis) after 12 months (dashed line) and 24 months (solid line), for both complete cases (orange) and single imputation nested in the bootstrap percentile method using 5,000 bootstrap replications (black).

**REFERENCES SUPPLEMENTARY APPENDIX**

1. Brand J, Van Buuren S, Le Cessie S, Van den Hout W. Combining multiple imputation and bootstrap in the analysis of cost-effectiveness trial data. Stat Med 2019;38(2):210-20.
2. Van Buuren S. Flexible imputation of missing data, second edition. Boca Raton: CRC Press 2018.
