# Supplementary figures and images for: Septoplasty versus non-surgical management for nasal obstruction in adults with a deviated septum: economic evaluation alongside a randomized controlled trial
Source: BMC Med. 2020 May 1;18:101. doi: 10.1186/s12916-020-01562-5 (PMC7193380; doi:10.1186/s12916-020-01562-5)

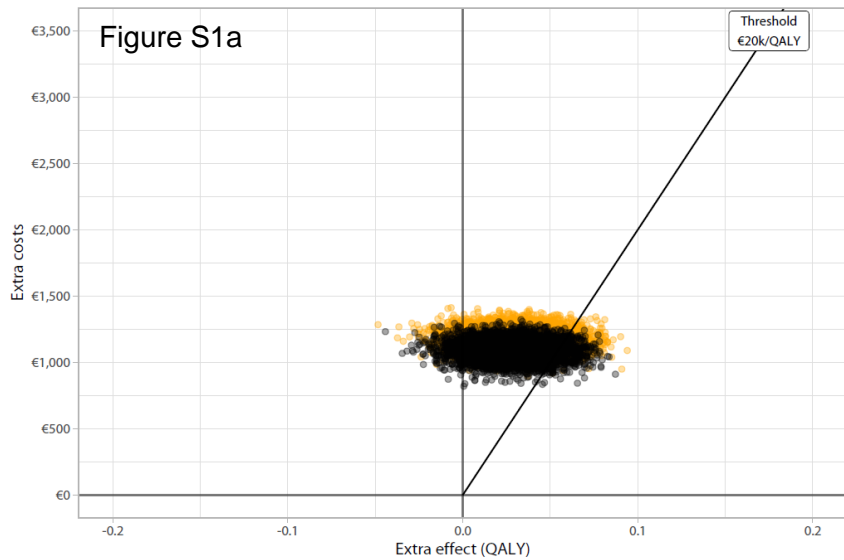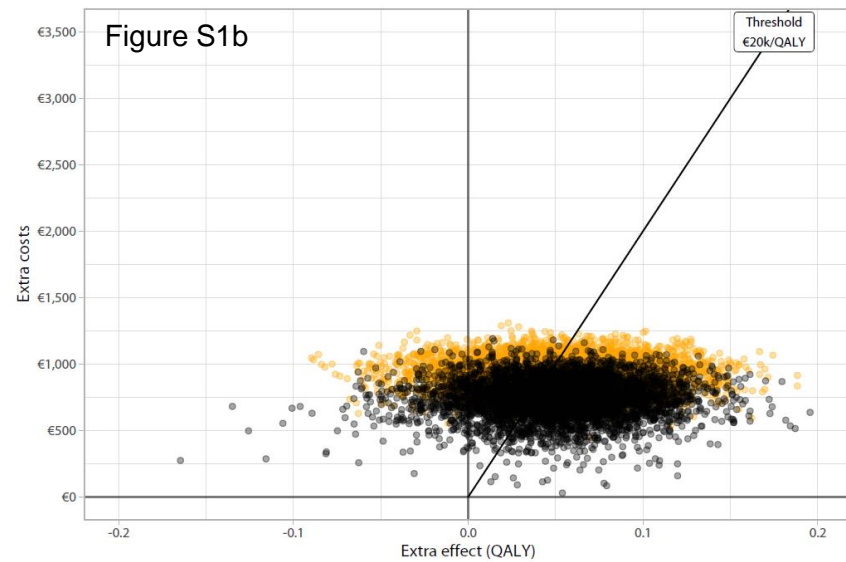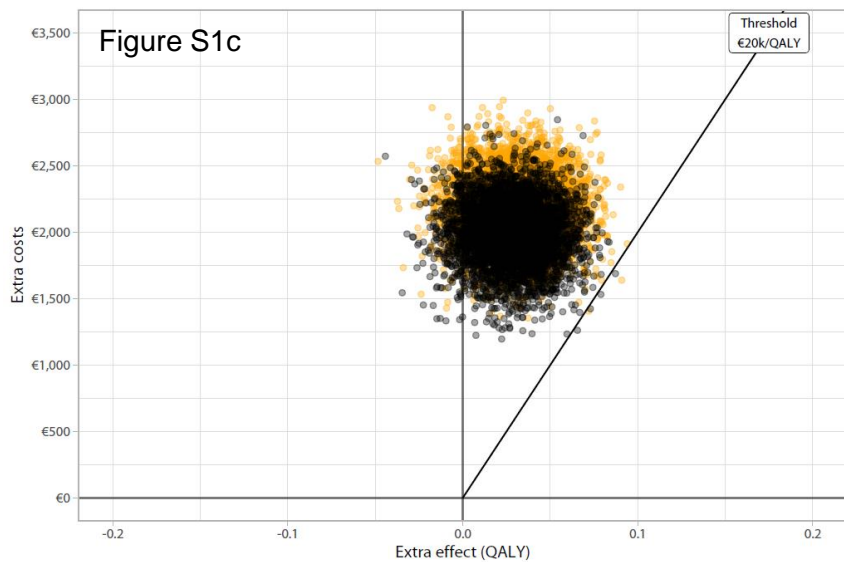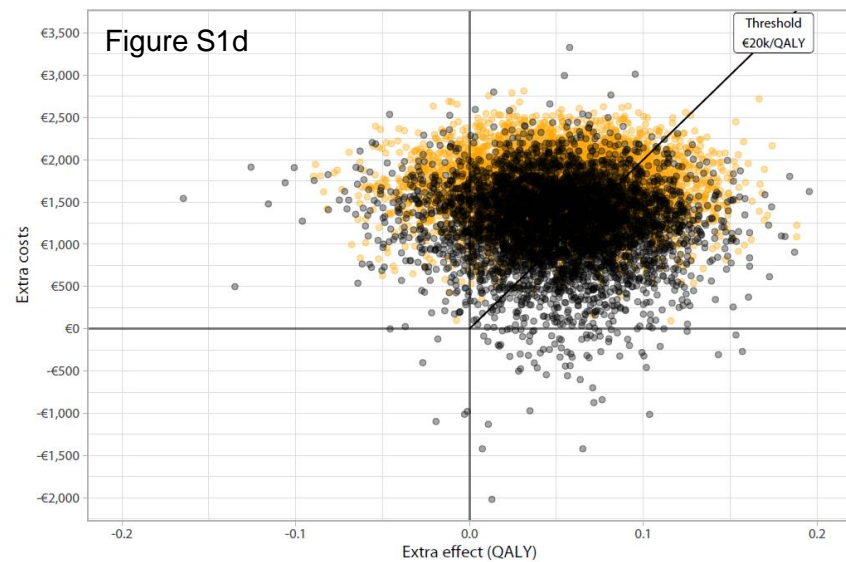

Supplement: Supplementary file 1 — Additional file 1. Missing data and single imputation nested in the bootstrap percentile method. [file 12916_2020_1562_MOESM1_ESM.zip › Figures S1a - S1d.pdf]

Figure S2a

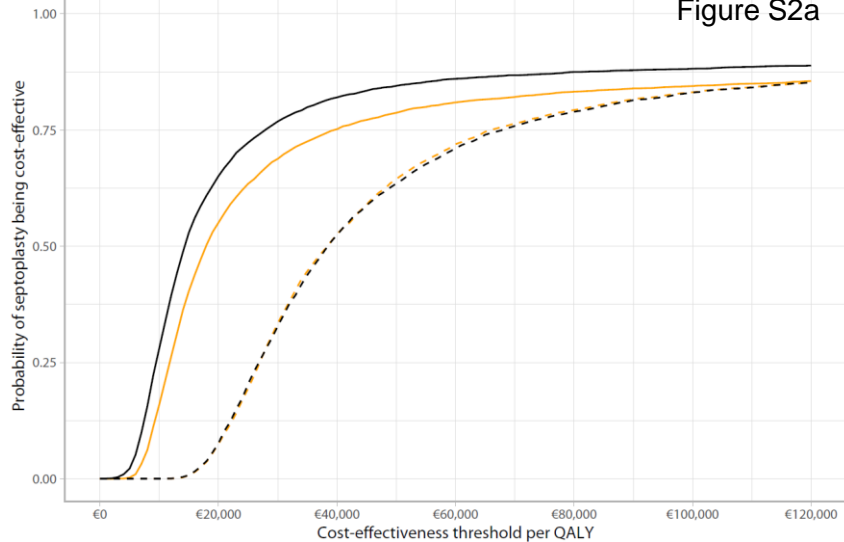

Figure S2b

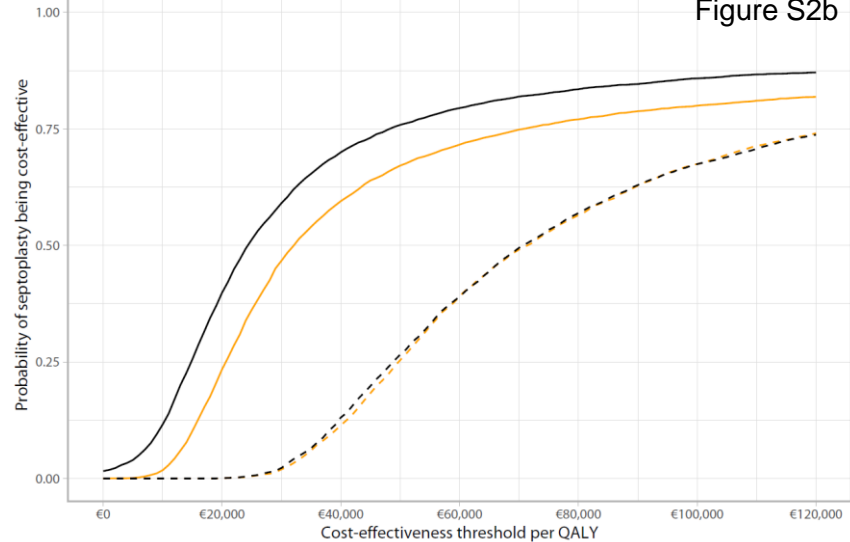

Supplement: Supplementary file 1 — Additional file 1. Missing data and single imputation nested in the bootstrap percentile method. [file 12916_2020_1562_MOESM1_ESM.zip › Figures S2a - S2b.pdf]
